# Platelet Membrane Biomimetic Nanoparticles Combined With UTMD to Improve the Stability of Atherosclerotic Plaques

**DOI:** 10.3389/fchem.2022.868063

**Published:** 2022-03-08

**Authors:** Jia Zhou, Chengcheng Niu, Biying Huang, Sijie Chen, Caigui Yu, Sheng Cao, Wenjing Pei, Ruiqiang Guo

**Affiliations:** ^1^ Department of Ultrasound Imaging, Renmin Hospital of Wuhan University, Wuhan, China; ^2^ Department of Ultrasound Medicine, The First Affiliated Hospital, Hengyang Medical School, University of South China, Hengyang, China; ^3^ Department of Ultrasound Diagnosis, The Second Xiangya Hospital, Central South University, Changsha, China

**Keywords:** ultrasound-targeted microbubble destruction (UTMD), rapamycin (RAP), biomimicking nanoparticles, atherosclerotic plaques, platelet membrane coating

## Abstract

Although research on the treatment of atherosclerosis has progressed recently, challenges remain in developing more effective, safer and transformative strategies for the treatment of atherosclerosis. Nanomaterials have recently played a unique role in many fields, including atherosclerosis treatment. Platelets are common component in the blood. Due to their inherent properties, platelets can target and adhere to atherosclerotic plaques. Ultrasound-targeted microbubble destruction (UTMD) shows great prospects in promoting the efficiency of drug delivery in treating solid tumors. In this study, we explored the possibility that UTMD assists platelet biomimetic rapamycin (RAP)-loaded poly (lactic-co-glycolic acid) (PLGA) nanoparticles (RAP@PLT NPs) in the treatment of atherosclerosis. The biomimetic nano-formulations exhibit better targeting ability to plaques when administered *in vivo*. Targeted destruction of Sonovue™ in the aortic area further improved the efficiency of targeting plaques. Moreover, the progression of atherosclerotic plaques was inhibited, and the stability of plaques was improved. Together, our study established a novel strategy for targeted delivery of nanoparticles in atherosclerotic plaques, by combining the advantages of the ultrasonic cavitation effect and biomimicking nanoparticles in drug delivery.

## Introduction

Atherosclerosis (AS) is a disease characterized by chronic inflammation of the arteries, and the main cause is disorders of lipid metabolism and the inflammatory response (Wang et al., 2021), which are mainly characterized by the thickening of arterial walls (Libby, 2012; Shapiro and Fazio, 2016; Moriya, 2019). The thickening and hardening of the arterial walls will narrow the arterial lumen, making it difficult for blood to flow through, causing various ischemic cardiovascular and cerebrovascular diseases.

Among atherosclerotic plaques, plaques with a high risk of rupture are called vulnerable plaques, while plaques with a lower risk of rupture are called stable plaques. Vulnerable plaques are usually characterized by thin fibrous caps, high lipid contents, increased lymphocytes, and increased apoptotic macrophages (Winkel et al., 2014). Sometimes the stenosis caused by vulnerable plaques is not very severe, but the risk of rupture, bleeding, thrombosis, or spasm is higher than that of stable plaques. Therefore, if the vulnerable plaque can be converted into a more stable plaque, it will make a positive difference in the clinical prevention of acute cardiovascular or cerebrovascular diseases.

In recent years, nanomedicine has been widely used for the targeted therapy of atherosclerosis (Yu et al., 2020; Costagliola di Polidoro et al., 2021; Craparo et al., 2021), but there are still challenges in developing a safe, effective and convertible nanoplatform. PLGA nanoparticles have been widely used in research due to their good stability, perfect biodegradability, and ideal biocompatibility (Hu et al., 2015; Wang et al., 2018; Pei et al., 2020). RAP is a new type of immunosuppressant with excellent antiproliferative ability. It is currently widely used clinically to avoid immune rejection after organ transplantation. Mammalian target of rapamycin (mTOR), which is the target of RAP is the core regulatory factor of cell homeostasis (Gaspari et al., 2006). By inhibiting the mTOR signaling pathway, RAP reduces inflammation, inhibits smooth muscle cell (SMC) proliferation and migration, and promotes autophagy and immune regulation. Therefore, RAP is considered for antiatherosclerosis treatment, but still has limitations due to formulation problems and poor bioavailability (Haeri et al., 2018). In addition, inhibition of the pathway regulated by mTOR induces other side effects, such as immunosuppression, dyslipidemia, and hyperglycemia (*via* oral administration) (Liu et al., 2019).

Cell membrane coating technology has attracted widespread attention (Hu et al., 2015), (Fang et al., 2018; Zhang et al., 2018). It promotes the efficiency of drug delivery, imaging and photoactivatable therapy, detoxification, and immune modulation (Hu et al., 2011; Fang et al., 2018). As it retains the natural membrane structure of cells, the wrapped nanoparticles will be endowed with a longer circulation time, lower immunogenicity, and selective targeting ability (Hu et al., 2016; Rao et al., 2018a; Bernal-Chávez et al., 2020). Platelets (PLT) are closely related to atherosclerosis (Kirschbaum et al., 2017; Li et al., 2018), and their inherent plaque adhesion properties will play an important role in the targeted delivery of nanodrugs to atherosclerotic plaques to improve therapeutic effects and reduce side effects (Song et al., 2019).

Ultrasound-targeted microbubble destruction (UTMD) is a promising noninvasive targeted drug delivery technology. It has shown great potential as a supporting strategy for targeted treatment of atherosclerotic plaques (Su et al., 2017; Yuan et al., 2018; Yang et al., 2019). UTMD is a process in which sound energy induces bubble collapse, generating microjets and creating transient holes in the cell membrane to promote the transport of macromolecules to the cytoplasm (Bekeredjian et al., 2003; Kinoshita and Hynynen, 2005; Tlaxca et al., 2010). If properly activated by ultrasound (US), intravascularly administered microbubbles can deliver DNA plasmids, siRNA, proteins, or conventional drugs to endothelial cells (Sun et al., 2019).

Hence, in this study, we used platelet membranes to coat RAP-loaded PLGA nanoparticles to prepare RAP@PLT NPs. We tested the characterization properties and explored the affinity with foam cells and macrophages. ApoE^−/-^ mice were fed with a high fat diet (HFD) for 12 weeks, and RAP@PLT NPs with SonoVue™ + US were applied in animal experiments. We found that RAP@PLT NPs + SonoVue™ + US can improve plaque stability and inhibit atherosclerotic plaques.

## Methods and Materials

### Materials

Poly (lactide-co-glycolide) (PLGA), polyvinyl alcohol (PVA) and IR780 iodide were all purchased from Sigma–Aldrich (United States). Rapamycin (RAP) was purchased from Dalian Meilun Biotechnology (China). DAPI, DiI and Cell Counting Kit-8 (CCK-8) were obtained from Beyotime Biotechnology (China). SonoVue™ was a gift from Bracco Imaging B.V. (Italy). RAW 264.7 cells were ordered from American Type Culture Collection (United States). Oxidized LDL was obtained from Yiyuan Biotechnologies (China). All experiments were approved by the Ethics Committee of the Second Xiangya Hospital, Central South University, China.

### Preparation of PLT Vesicles and RAP@PLT NPs

The PLT vesicles obtained from 1 ml of whole blood of mice were prepared as described previously (Rao et al., 2017) and used to cloak 1 mg of RAP@NPs (Supporting information). The extra PLT vesicles were removed (12,000 rpm, 10 min), then washed and sonicated using a bath sonicator (53 kHz, 100 W) for 5 min. Then, 1 ml of 1 × PBS containing 1 mg of RAP@NPs was mixed with the PLT vesicles and sonicated for 30 s to complete the membrane coating. The collected RAP@PLT NPs were redispersed in 1 × PBS and kept at 4°C.

### Characterization of RAP@PLT NPs

The size and surface potential of RAP@PLT NPs were analyzed using a dynamic light scattering (DLS) analyzer (Malvern Nano ZS, United Kingdom). The structures of PLT vesicles, RAP@NPs and RAP@PLT NPs were examined by transmission electron microscopy (TEM, G2 F20 S-TWIN, Tecnai). Briefly, the samples were placed on a carbon coated copper grid and stained with 3% (w/v) uranyl acetate for TEM viewing. The membrane protein analysis of fresh PLTs, PLT vesicles and RAP@PLT NPs was characterized by Western blot. The protein of fresh PLTs, PLT vesicles and RAP@PLT NPs was extracted and the protein concentration was quantified by a BCA protein assay kit. The key membrane proteins were detected using primary antibodies including rabbit anti-mouse CD47 antibody; and rabbit anti-mouse Integrin β1 antibody. All samples were run at the same total protein concentration. To understand the stability of RAP@PLT NPs, the DLS diameters were measured at preset time points in 48 h in 10% fetal bovine serum (FBS).

### Drug Loading and Encapsulation Characterization

Ten milligrams, 5 mg or 3 mg of RAP was loaded onto uncoated RAP@NPs in a similar procedure. The drug loading and encapsulation efficiency of RAP were measured by high-performance liquid chromatography (HPLC). According to the pre-established standard curve of RAP in acetonitrile, the RAP encapsulation efficiency (EE) and loading efficiency (LE) were calculated as follows (Wang et al., 2021) and (Libby, 2012):

$$\text{EE}\left(\text{\%}\right)=\frac{W_{R}}{W_{T}}\times 100\text{\%}$$ (1)

$$\text{LE}\left(\text{\%}\right)=\frac{W_{R}}{W_{NPS}}\times 100\text{\%}$$ (2)

in which $W_{R}$ is the weight of RAP loaded in RAP@NPs, $W_{T}$ is the weight of the total added RAP, and $W_{NPs}$ is the weight of lyophilized powder of RAP@NPs.

### *In Vitro* Drug Release

To evaluate the drug release profile, *in vitro* RAP release tests of RAP@NPs and RAP@PLT NPs with or without SonoVue™ microbubble + US irradiation were studied, Briefly, RAP@NPs and RAP@PLT NPs solutions (10 mg/ml, 1 ml) diluted with PBS or SonoVue™ were added to dialysis bags (Mw = 8,000 Da); and placed in a glass bottle with 20 ml of PBS, and groups containing SonoVue™ microbubbles were irradiated by an US transducer (1 MHz, 2 W/cm^2^, 40% duty cycle. WED-100, WELLD Medical Electronics, China) for 5 min. Then, each group was placed in a shaker (37°C, 100 rpm). At predetermined time points, 0.5 ml of supernatant was removed, and the same volume of fresh PBS was replenished. The RAP concentration was quantified by high-performance liquid chromatography (HPLC).

### Cell Culture

The RAW264.7 cells were cultured and maintained in DMEM containing 10% (v/v) FBS. To obtain foam cells, RAW264.7 cells were cultured in the presence of 100 μg/ml oxidized low-density lipoprotein (LDL) for 48 h, washed with PBS, and stained with Oil Red O to confirm successful conversion. Under bright field observation, the red substance appeared in cells, confirming that the foam cells were successfully prepared.

### Cytotoxicity Evaluation

RAW264.7 macrophages were cultured in 96-well plates at a density of 1.0 × 10^4^ cells per well. Cells were incubated at 37 °C in a humidified atmosphere containing 5% CO_2_ for 24 h. Then, cells were treated with fresh DMEM containing free RAP, uncoated nanoparticles and platelet membrane-coated RAP@PLT NPs at various doses. After different time periods, cell viability was quantified by CCK-8 assay.

### Nanoparticles Uptake by Foam Cells

Foam cells were transformed from RAW264.7 cells by oxidized low-density lipoprotein (LDL). After confirming successful conversion, DiI@PLGA NPs and PLT-DiI@PLGA NPs were added and incubated for 2 h. Then, the cells were gently washed with PBS and fixed with 4% paraformaldehyde. The cells were stained with DAPI and examined by inverted fluorescence microscopy. ImageJ was used for quantitative analysis.

### Animals Model

Male C57BL/6 mice and male apolipoprotein E-deficient ApoE^−/−^ mice (25–30 g, eight-weeks old) were obtained from the Medical Experimental Animal Center of the Second Xiangya Hospital, Central South University (Changsha, China). All animal experiments were approved by the Ethics Committee of the Second Xiangya Hospital of Central South University and conducted in accordance with the guidelines of the Department of Laboratory Animals of Central South University. Atherosclerosis model mice were prepared by feeding ApoE^−/−^ mice a high-fat diet (HFD) for 12 weeks.

### Blood Circulation Time and *in Vivo* Distribution

The pharmacokinetics study was carried out by using adult male C57BL/6 mice weighing 25 ± 2 g. IR780 was used as fluorescent indicator to track the nanoparticles. Briefly, IR780 labeled-RAP@NPs and RAP@PLT NPs were injected intravenously (200 μl, 20 mg/ml of NPs, IR780 of 0.5 mg/ml), and 50 μl of whole blood was immediately collected from the submandibular vein after 1, 5, 15, 30 min, and 1, 2, 4, 6, 18, 24, and 48 h. Blood samples were diluted, and the plasma was separated and added in 96-well plates. The absorbance of IR780 was measured with a microplate reader (TECAN M1000, United States) to quantify the concentration.

To confirm the *in vivo* distribution of RAP@PLT NPs, IR780 was also used to label nanoparticles as a fluorescent imaging agent. Two hundred microliters of uncoated IR780 labeled RAP@NPs and PLT-coated RAP@PLT NPs were injected into C57BL/6 mice intravenously *via* the tail vein. After 24 h, the mice were sacrificed, the main organs were removed for imaging, and the average fluorescence intensity of the region of interest (ROI) was quantified by Living Image.

### Accumulation of RAP@PLT NPs in Atherosclerotic Plaques

Atherosclerosis model mice were divided into five groups and intravenously injected with PBS, IR780-labeled RAP@NPs, RAP@PLT NPs, RAP@NPs + SonoVue™ and RAP@PLT NPs + SonoVue™, and the RAP@NPs + SonoVue™ and RAP@PLT NPs + SonoVue™ groups were irradiated with US for 30 s (2 W/cm^2^). After 2 h, mice were euthanized, perfused with PBS containing 4% paraformaldehyde and heparin sodium, and the aortas were removed, and the fluorescence of each group was observed by a Lumina IVIS Spectrum imaging system (PerkinElmer, United States). The average fluorescence intensity of the region of interest (ROI) was quantified by Living Image.

### Treatment for Atherosclerotic Mice

ApoE^−/−^ mice were randomized into seven groups (*n* = 5) and received a HFD for 12 weeks. Then, we give different treatments to those mice every 2 days for a month, those which injected with saline were served as the control group, while the other five groups were treated with free drug (RAP of 0.7 mg/kg), RAP@NPs (RAP of 0.7 mg/kg), RAP@PLT NPs (RAP of 0.7 mg/kg), RAP@NPs + SonoVue™ + US (RAP of 0.7 mg/kg) or RAP@PLT NPs + SonoVue™ + US (RAP of 0.7 mg/kg). The US groups were irradiated by an US transducer (1 MHz, 2 W/cm^2^, 40% duty cycle,WED-100, WELLD Medical Electronics, China), the power density of US was 2 W/cm^2^ for 30 s, the injection dosage of SonoVue™ ^(^5 mg/ml) was 100 μl each mouse. SonoVue™ was fully mixed with RAP@NPs or RAP@PLT NPs when administered to ApoE^−/−^ mice. The US transducer was imposed at the chest of ApoE^−/−^ mice. The body weight of those mice was recorded every other day.

### Detection and Quantify of Atherosclerotic Plaques

After the 30 days treatment, the aortas of ApoE^−/−^ mice were harvested and fixed with paraformaldehyde (4% in PBS) by perfusion from heart to iliac bifurcation. After carefully peeling the periadventitial tissue off the aortas, we longitudinally dissected and stained with Oil Red O (ORO) to observe the plaque area. ORO staining of the slices of the aortic root was further developed to determine the extent of atherosclerosis at the aortic root. Image-Pro Plus 6.0 software was used to analyze the atherosclerotic plaque areas.

### Histology and Immunohistochemistry Analysis

The aortic roots of ApoE^−/−^ mice treated for 30 days were fixed (with 4% paraformaldehyde) and prepared into paraffin sections. Then, Masson’s trichrome and toluidine blue staining were performed to show the of collagen and necrotic cores. Immunohistochemical staining was performed to quantify macrophages, MMP-9, smooth muscle cells (SMCs), or endothelial cells (EC). The sections were processes according to the usual procedures and incubated with antibodies against CD68, α-smooth muscle actin (α-SMA), matrix metalloproteinase-9 (MMP-9) or CD31. Finally, Image-Pro Plus 6.0 software was used for quantitative analysis. Then the sections of aortic roots and the main organs (heart, liver, spleen, lung, kidney and brain) were analyzed by hematoxylin-eosin (HE) staining.

### Statistical Analysis

The data were showed as the mean ± s. d. and analyzed by using GraphPad Prism version 6.0 software (GraphPad, United States). Comparisons between two groups were made by Student’s *t* test, and the significant differences among the groups were carried out by one-way analysis of variance (ANOVA) by Tukey’s test. The significance levels of the differences were set to *p < 0.05, **p < 0.01 and ***p < 0.001.

## Results

### Preparation and Characterization of RAP@PLT NPs

**FIGURE 1:**
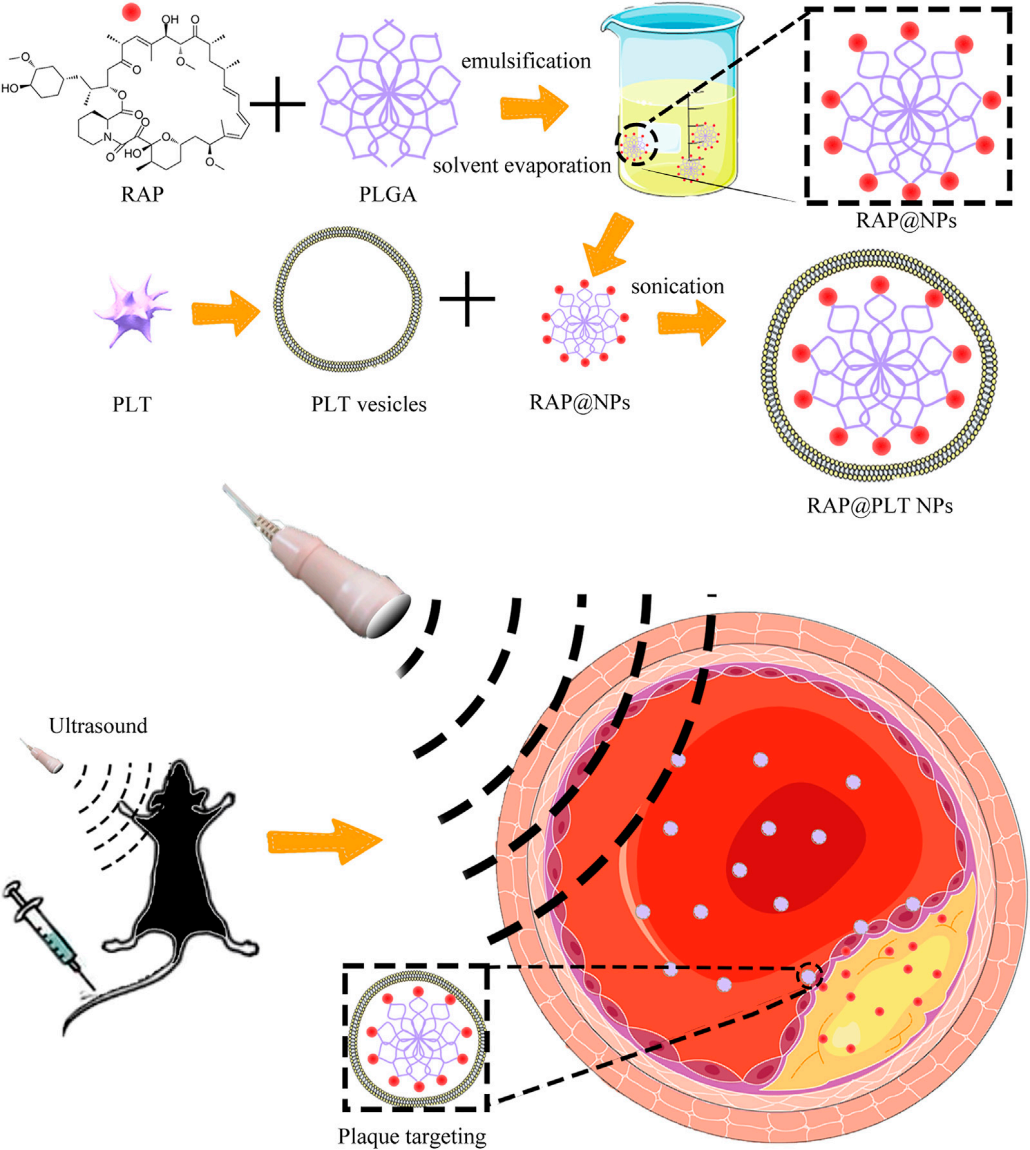
The illustration of the preparation steps of RAP@PLT NPs and the working mechanism *in vivo*.

**FIGURE 2:**
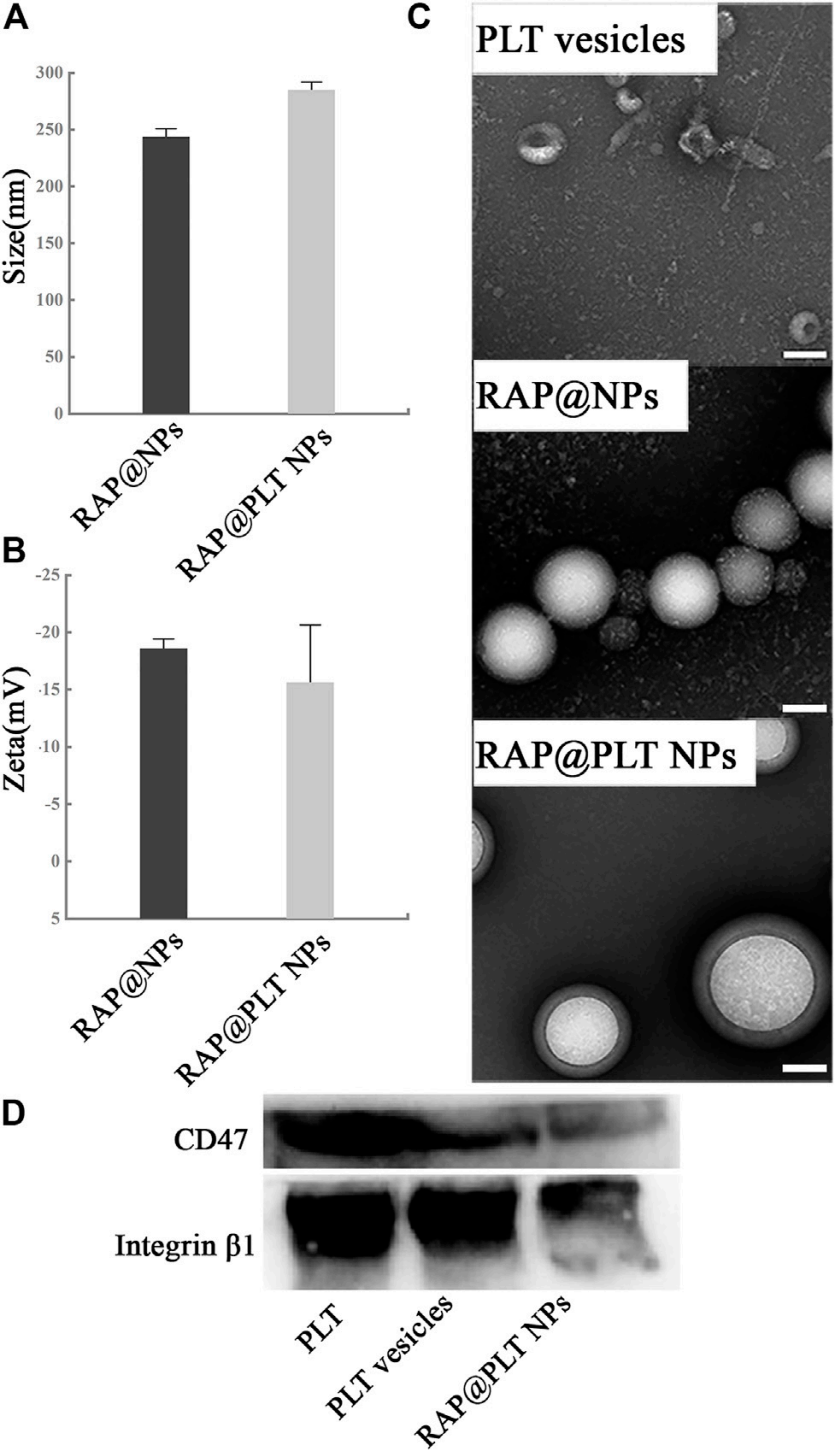
Characterization of RAP@PLT NPs. **(A)** Size and **(B)** Zeta potential of RAP@NPs and RAP@PLT NPs (before and after coating with PM). **(C)**The TEM image of platelet vesicles. Scale bar: 100 nm. **(D)** Representative CD47 and Integrin β1 protein bands of fresh PLT, PLT vesicles and RAP@PLT NPs resolved using Western blotting.

To form RAP@NPs, we used the previous single emulsification-solvent volatilization method to load hydrophobic RAP onto PLGA nanoparticles (Wang et al., 2018; Pei et al., 2020). Then, we used the repeated freeze-thaw method (Rao et al., 2017; Rao et al., 2018b) to obtain platelet membrane vesicles and prepare RAP@PLT NPs (Figure 1). Dynamic light scattering (DLS) was employed to measure the size of RAP@NPs before and after wrapping with PLT membrane vesicles. Surface wrapping of the PLT membrane increased the hydrodynamic diameter of RAP@NPs from 256 to 287 nm (Figure 2A). The zeta potential decreased from −18.5 mV to −15.6 mV (Figure 2B). The larger size and higher potential of the new formulation demonstrate the successful acquisition of RAP@PLT NPs. The diameter of the formulation was smaller than the capillaries, so the RAP@PLT NPs could be delivered to the whole blood circulation, and occlusion would not occur. As shown in Figures 2A,C thick membrane layer was observed after coating with the PLT membrane by using TEM. The nanoparticles showed a uniform size and core-shell structure, and the thickness of the membrane layer was approximately 31 nm. Furthermore, RAP@PLT NPs showed a comparatively constant hydrodynamic diameter in FBS (37°C), indicating their excellent colloidal stability (Supplementary Figure S1). To certify the presence of the platelet membrane on the nanoparticles, we used Western blotting to detect the protein content, including CD47 and integrin β1, on fresh platelets, platelet vesicles and RAP@PLT NPs. The immunomodulatory protein CD47 could send signals such as “Don’t eat me” to macrophages (Gardner et al., 2020), which could further help RAP@PLT NPs escape from the immune system. As shown in Figure 2D, protein bands of both CD47 and integrin β1 appeared, which indicated the existence of a platelet membrane. The expression of CD47 in the PLT, PLT vesicles and RAP@PLT NPs were 100, 85.7 and 60.2%, respectively. The expression of integrin β1 in the PLT, PLT vesicles and RAP@PLT NPs were 100, 97.4 and 79.2%, respectively.

RAP is poorly dissolved in water, and the saturation solubility in water is only 2.6 μg/ml (Simamora et al., 2000). HPLC was applied to examine the content of RAP in RAP@NPs. We adapted 3 protocols to determine the best one for RAP loading. As shown in Supplementary Figure S2, when 100 mg of PLGA and 3 mg of RAP were added to the organic phase, the drug encapsulation efficiency (EE) was the highest when compared with the other two protocols. Therefore, in subsequent experiments, we adapted this ratio to prepare RAP@NPs.

To understand the RAP release kinetics of RAP@NPs and RAP@PLT NPs, we carried out an *in vitro* experiment to simulate the situation in blood circulation. As shown in Supplementary Figure S3, after shaking gently for 72 h in a shaker, 42.12% of RAP was released from RAP@NPs, while 33.74% of RAP was released from RAP@PLT NPs. After US irradiation, the RAP release was increased to 75.57 and 67.54% of RAP@NPs and RAP@PLT NPs, respectively. The overall RAP release trend of RAP@PLT NPs was slower, which may contribute to the coating of the platelet membrane becoming a barrier to decelerate the rapid release of RAP. In addition, it is noteworthy that with US irradiation after mixing with SonoVue™ microbubbles, the efficiency of RAP release was increased from both RAP@NPs and RAP@PLT NPs.

### Cytotoxicity Research of RAP@PLT NPs

Considering that macrophages are closely interrelated to the occurrence and progression of atherosclerosis, we tested the cytotoxicity of RAP@PLT NPs in suppressing the proliferation of RAW 264.7 cells by comparison with free RAP and RAP@NPs. In Supplementary Figure S4, we set a series of concentration gradients to compare the effects of free RAP and both nanoformulations to suppress the proliferation of macrophages. Free RAP inhibited RAW264.7 cells in a concentration-dependent pattern, while RAP@NPs and RAP@PLT NPs had less impact on the proliferation of RAW264.7 cells, which may be attributed to the structure of RAP@NPs and RAP@PLT NPs with PLT membrane coating, leading to slower RAP release and milder cytotoxicity.

### Cellular Uptake of RAP@PLT NPs by RAW264.7

*In vitro* cellular uptake of RAP@PLT NPs by RAW264.7 cells was observed using an inverted fluorescence microscope. We used DiI-labeled PLGA nanoparticles to conveniently track the phagocytic activity and compare the amount of fluorescence in the PLT membrane coated group and the uncoated group. We encapsulated DiI dyes into PLGA nanoparticles at the initial steps by simply fusing to synthesize DiI@PLGA. After that, the PLT membrane was coated onto DiI@PLGA to form PLT-DiI@PLGA, so the concentration of DiI dyes was the same in both groups. The fluorescent images of PLT-DiI@PLGA around RAW264.7 cells showed weaker fluorescence compared to DiI@PLGA at 2.0 h, suggesting that endocytosis of RAP@PLT NPs was less than that of the uncoated group at 2.0 h. The results of fluorescence quantitative analysis showed that the uncoating was nearly 3-fold greater than the coating (***p < 0.001), suggesting that PLT membrane coating can prevent the clearance of macrophages, so the disguise of RAP@PLT NPs was successful and improved the biocompatibility of nanoparticles.

### Cellular Uptake of RAP@PLT NPs by Foam Cells

**FIGURE 3:**
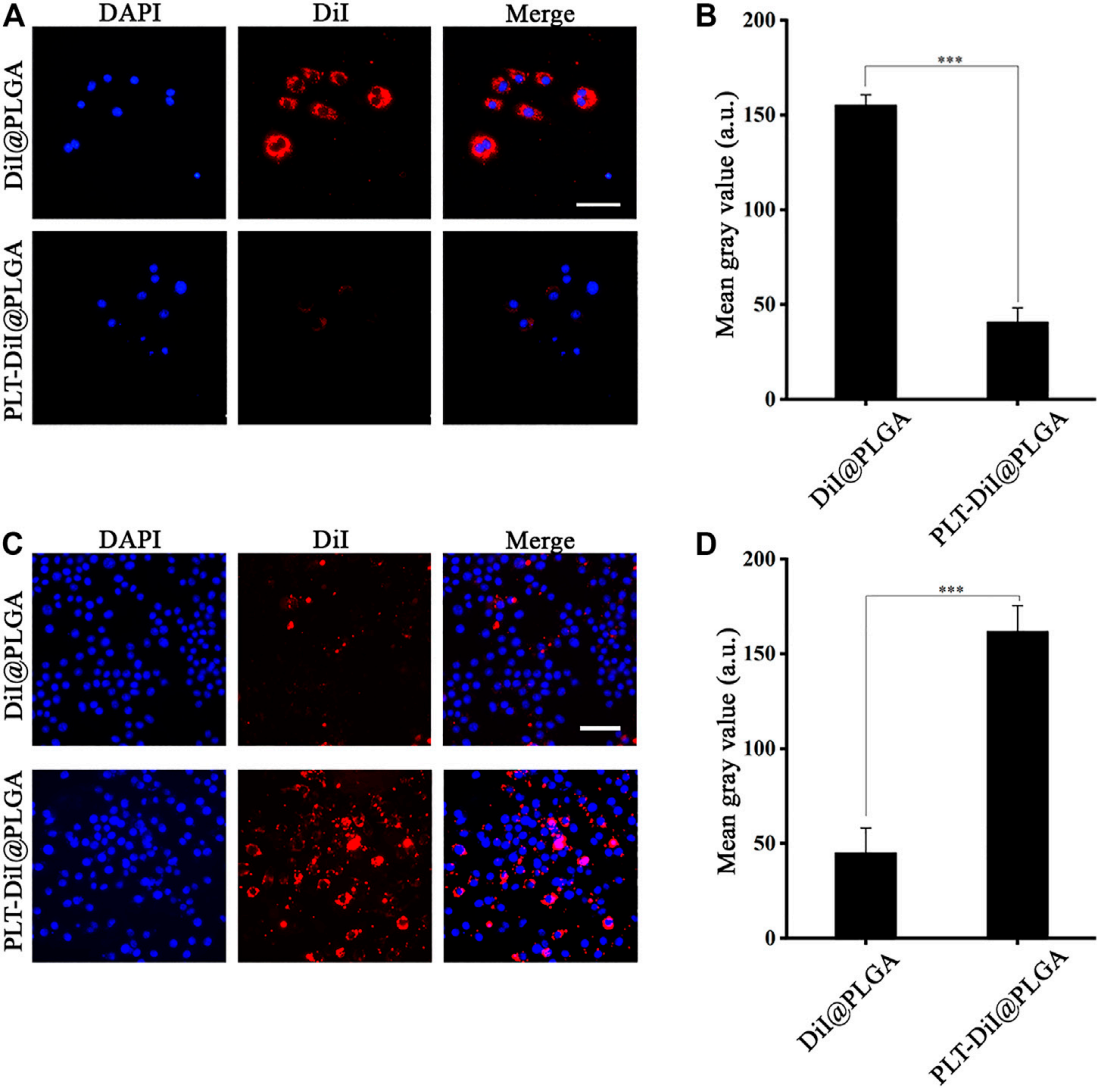
The Fluorescence imaging of the RAW264.7 and foam cells. **(A)** Fluorescence images of accumulated DiI@PLGA and PLT-DiI@PLGA in RAW264.7 cells. Scale bar = 100 µm. **(B)** Corresponding fluorescence intensities of RAW264.7 cells treated with DiI@PLGA and PLT-DiI@PLGA for 2.0 h (***p < 0.001). **(C)** Fluorescence images of accumulated DiI@PLGA and PLT-DiI@PLGA in foam cells. Scale bar = 50 µm. **(D)** Corresponding fluorescence intensities of foam cells treated with DiI@PLGA and PLT-DiI@PLGA for a 2.0 h (***p < 0.001).

Macrophages convert into foam cells after they phagocytose excessive lipids at plaque sites. As shown in Supplementary Figure S5, we successfully obtained foam cells by transformation. We assumed that nanoformulations with the ability to target atherosclerotic plaques could improve the therapeutic effect by better releasing RAP at plaque sites. RAP@PLT NPs will adhere to the plaques inherently due to the existence of platelet membranes, as platelets have inherent affinity to atherosclerotic plaques, and they could naturally home to the plaque sites. As shown in Figures 3A–D, compared with DiI@PLGA, PLT-DiI@PLGA showed more fluorescence around the foam cells at the same time point, which proved that the coating of the platelet membrane could efficiently enhance the targeting ability of RAP@PLT NPs to foam cells.

### Targeting Ability to Atherosclerotic Plaques *in Vivo*

**FIGURE 4:**
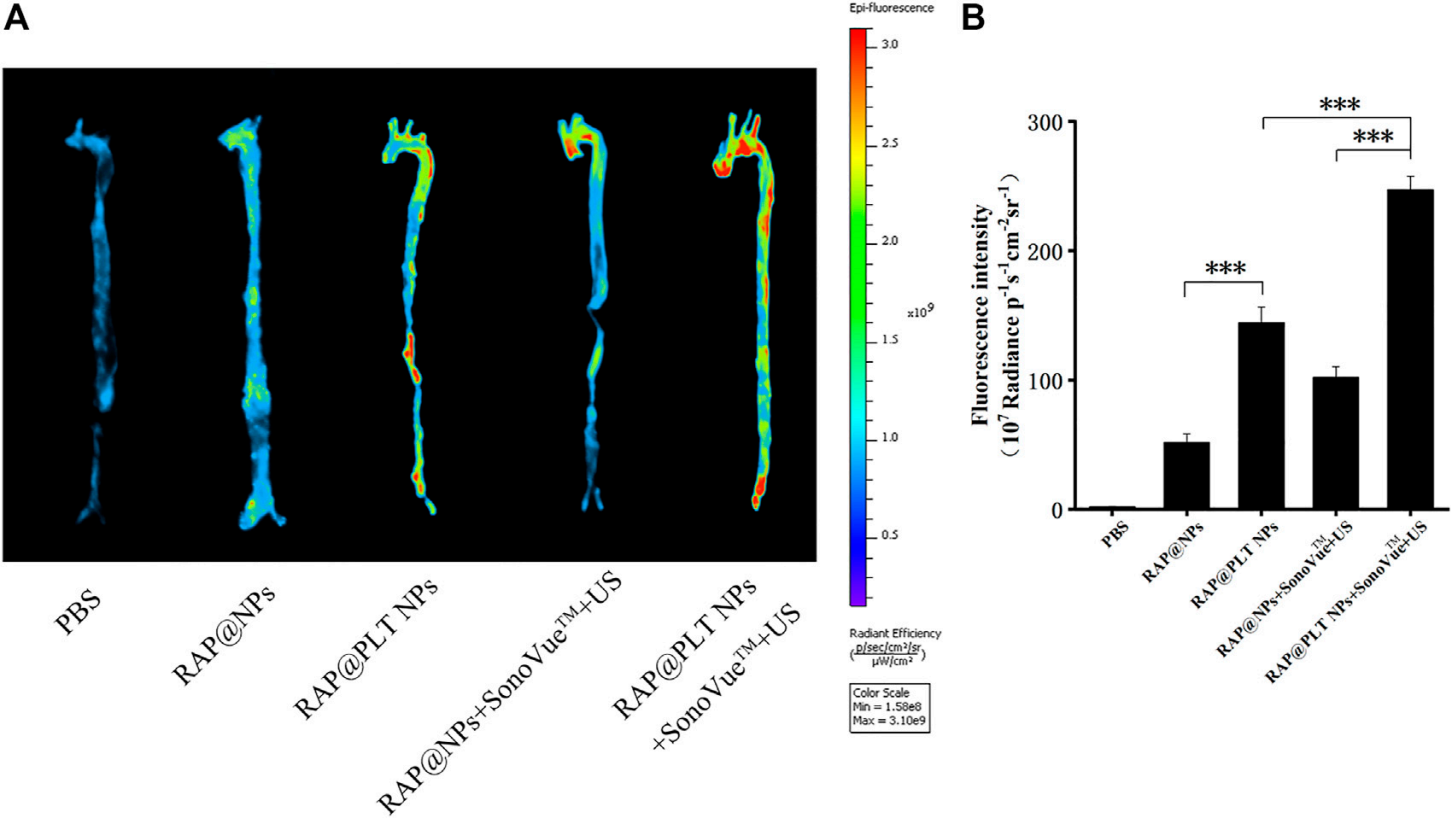
The Fluorescence imaging of the aorta. **(A)** The *in vivo* fluorescence images of the aorta. **(B)** quantitative data of fluorescence signals accumulated in the aorta of ApoE^−/−^ mice after treatment of PBS, IR780-labeled RAP@NPs, RAP@PLT NPs, RAP@NPs + SonoVue™ + US and RAP@PLT NPs + SonoVue™ + US. *n* = 3, mean ± SD, *p < 0.05 and ***p < 0.001.

To investigate whether the joint design can increase the arrival of nanoparticles in the atherosclerotic plaque sites and maximize the benefits. We prepared ApoE^−/−^ mice on a HFD for 12 weeks. After intravenous injection of different nanoformulations or the mixture with SonoVue™ *via* the tail vein; and irradiation with US (2 W/cm^2^,30 s), the mice were killed after 2 h, the aortas were removed, and the fluorescence of each group was observed. As shown in Figures 4A,B, the fluorescence in RAP@PLT NPs groups was stronger than which in RAP@NPs groups, and after treated with SonoVue™ + US, the fluorescence became strongest in three groups, 1.71-folds greater than which in RAP@NPs groups (***p < 0.001), suggesting that more nanoparticles could adhere to the atherosclerotic plaques in the RAP@PLT NPs + SonoVue™ + US groups. We considered it related to the inherent plaque adhesion properties of PLT membrane, there were more RAP@PLT NPs adhered to the plaque site. After treated with SonoVue™ + US, the ultrasonic cavitation effect makes the microbubbles burst, the transient holes in the cell membrane open, and more nanoparticles could enter the atherosclerotic plaque sites, so that the display of fluorescence would be stronger.

### Blood Circulation Time and Biodistribution of RAP@PLT NPs *in Vivo*

To understand the metabolism of nanoparticles in the blood circulation, we used IR780-labeled RAP@NPs and RAP@PLT NPs to study the pharmacokinetics. As shown in Supplementary Figure S6, RAP@PLT NPs were cleared slower from the blood. Only 29.84% of RAP@NPs were still detected at 24 h, while 35.13% of RAP@PLT NPs could be detected. The half-life and clearance rate of the two were significantly different (Supplementary Table S1, supporting information), suggesting that the PLT membrane coating could keep nanoparticles in the blood circulation longer. Disguise of the PLT membrane avoided phagocytosis of the reticuloendothelial system. Therefore, the coating of the PLT membrane endowed nanoparticles with a stronger ability in targeted drug delivery.

As shown in Supplementary Figure S7, IR780-labeled RAP@NPs and RAP@PLT NPs both gathered in the lungs of mice, and this result may be connected with the characteristics of IR780 (Rajendrakumar et al., 2018). After PM-membrane coating, the intensity of the fluorescence in lungs was weaker than before (*p* < 0.01**), and the distribution of fluorescence in other organs showed no obvious significant difference.

### Targeted Ultrasonic Therapy of Atherosclerosis

**FIGURE 5:**
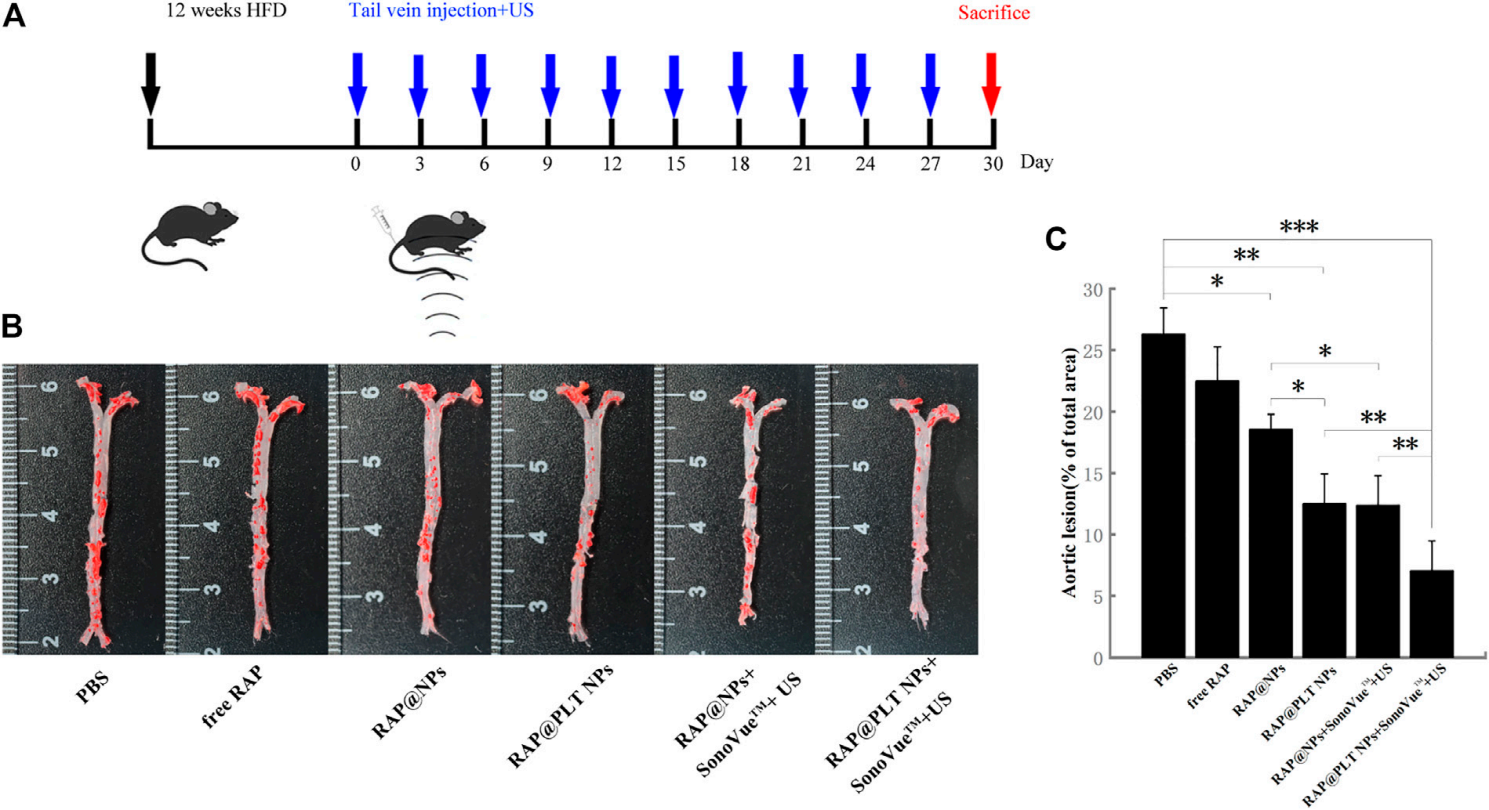
Atherosclerosis treatment in ApoE^−/−^ mice. **(A)** The illustration of various treatment in 30 days. **(B)** The en face ORO-stained images of aortas from each group. **(C)** Quantitative data of the atherosclerotic plaque area.

*In vivo* therapy was carried out in an atherosclerosis model using ApoE^−/−^ mice (Figure 5A). As shown in Figure 5B, after various treatments, the aortas of ApoE^−/−^ mice were only injected with saline that was intravenously distributed with the most atherosclerotic plaques, accounting for approximately 26.30% (Figure 5C). After treatment with free RAP, the plaques were slightly inhibited, and the plaque area was approximately 22.50%, which may be attributed to the hydrophobicity of RAP. RAP@NPs efficiently inhibited plaque progression as the plaque area decreased to approximately 18.57%. The targeted inhibition of RAP@PLT NPs significantly reduced the area of plaques to 12.53%. When treated with the SonoVue™ + US groups, the effects of inhibiting the development of plaques were significantly better than those groups without SonoVue™ + US. The plaque area in RAP@PLT NPs + SonoVue™ + US group was 7.05%.

**FIGURE 6:**
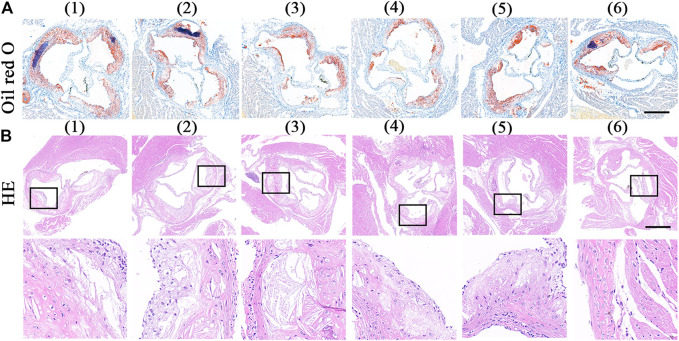
ORO and HE staining of sections of aortic roots. **(A)** the ORO-stained images of aortic roots sections (Wang et al., 2021).- (Costagliola di Polidoro et al., 2021) referred to (Wang et al., 2021) PBS (Libby, 2012); free RAP (Shapiro and Fazio, 2016); RAP@NPs (Moriya, 2019); RAP@PLT NPs (Winkel et al., 2014); RAP@NPs + SonoVue™ + US (Costagliola di Polidoro et al., 2021); RAP@PLT NPs + SonoVue™ + US; scale bar = 500 µm. **(B)** the HE staining sections of aortic roots sections and the enlarged version of the plaque area; scale bar = 500 µm.

**FIGURE 7:**
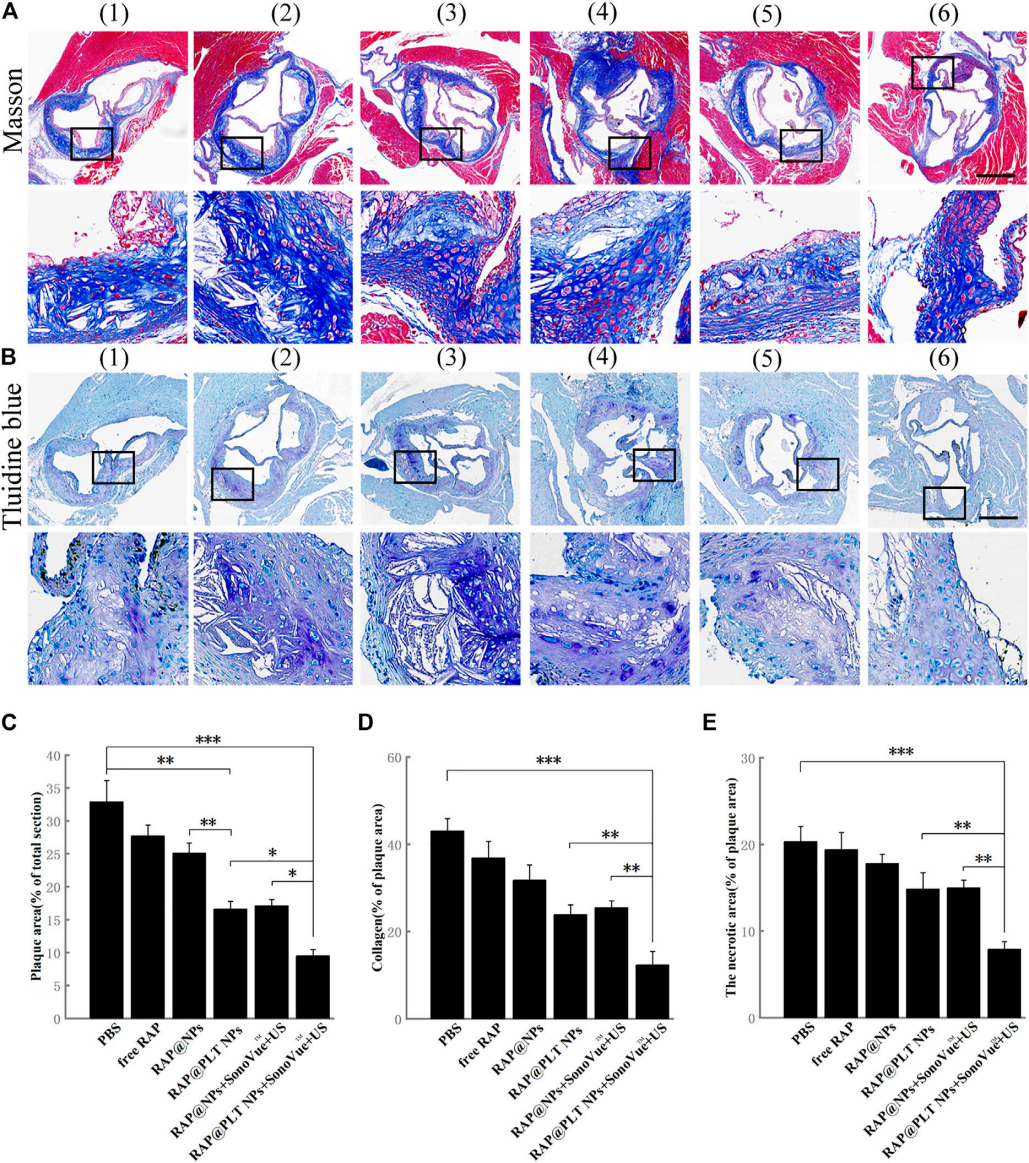
Immunohistochemistry analyses of aortic root sections from ApoE^−/−^ mice after different treatments. **(A)** The images of collagen in the plaque areas stained by Masson’s trichrome (scale bar = 500 µm). **(B)** The images of the necrotic areas stained by Toluidine blue (scale bar = 500 µm) (Wang et al., 2021).- (Costagliola di Polidoro et al., 2021) referred to (Wang et al., 2021) PBS (Libby, 2012); free RAP (Shapiro and Fazio, 2016); RAP@NPs (Moriya, 2019); RAP@PLT NPs (Winkel et al., 2014); RAP@NPs + SonoVue™ + US (Costagliola di Polidoro et al., 2021); RAP@PLT NPs + SonoVue™ + US. **(C)** Quantitative data of the atherosclerotic plaque area in the aortic root sections. **(D)** Quantitative data of the content of collagen in aortic root sections. **(E)** Quantitative data of the necrotic areas in the aortic root sections. *n* = 5, mean ± SD, *p < 0.05, **p < 0.01, and ***p < 0.001.

The extent of aortic plaques showed a similar trend with Oil Red O slice staining. As shown in Figure 6A, there were plenty of Oil Red O-marked lipids deposited in the plaques. Compared with the value of 32.91% in the control group, the average area of plaque decreased to 27.74, 25.12, and 16.62% after treated with free RAP, RAP@NPs and RAP@PLT NPs, respectively (Figure 7C). In addition, the raised plaques were smaller. The data decreased heavily in the RAP@PLT NPs + SonoVue™ + US group. These results suggest that RAP@PLT NPs + SonoVue™ + US can effectively attenuate the progression of atherosclerosis.

As shown by the result of H&E staining (Figure 6B), sections from the control group showed more acellular cores and cholesterol clefts than other groups, which were characteristics of complex lesions. After different treatments, the acellular cores were successively decreased. There were few acellular cores in the RAP@PLT NPs + SonoVue™ + US group.

Collagen was produced by proliferative SMCs. In the normal vascular wall, collagen was regular and completely distributed, while in atherosclerotic plaques, collagen was distributed in a disorderly manner and became thicker. The overaccumulation of collagen could lead to an increase in plaque area. As shown in Figures 7A,D, in the control group, the collagen content of the total valve section was 43.04%, and the data decreased to 36.87, 31.75, and 23.89% after various treatments, including free RAP, RAP@NPs and RAP@PLT NPs. This number further decreased after treatment with both nanoparticles with SonoVue™ + US, suggesting that the RAP@NPs can release RAP in the plaque sites; and inhibit the progression of atherosclerosis. The platelet membrane can enable more RAP to be accurately located on the plaque site. The ultrasonic cavitation effect allows more RAP to enter the plaque area and improves the treatment effect. The above results revealed that RAP@PLT NPs + SonoVue™ + US could obviously inhibit the progression of atherosclerotic plaques and decrease lipid deposition and collagen levels.

The necrotic areas were detected by toluidine blue staining. As shown in Figures 7B,E, large necrotic areas with substantial cholesterol crystals could be seen in the control group. After treatment with RAP@PLT NPs + SonoVue™ + US, the necrotic area was significantly decreased and the result was further proved by quantitative analysis. When compared to the control group, the necrotic area was decreased to 19.39, 17.81, and 14.86% in response to free RAP, RAP@NPs, and RAP@PLT NPs treatment. With the assistance of SonoVue™ + US, the necrotic area was further decreased to 14.99 and 7.91% in the RAP@NPs + SonoVue™ + US and RAP@PLT NPs + SonoVue™ + US groups. The above results indicate that RAP@PLT NPs + SonoVue™ + US could reduce the amount of MMP-9 and the necrotic areas of plaques; thus, the possibility of plaque rupture was decreased, and plaque stability was improved.

**FIGURE 8:**
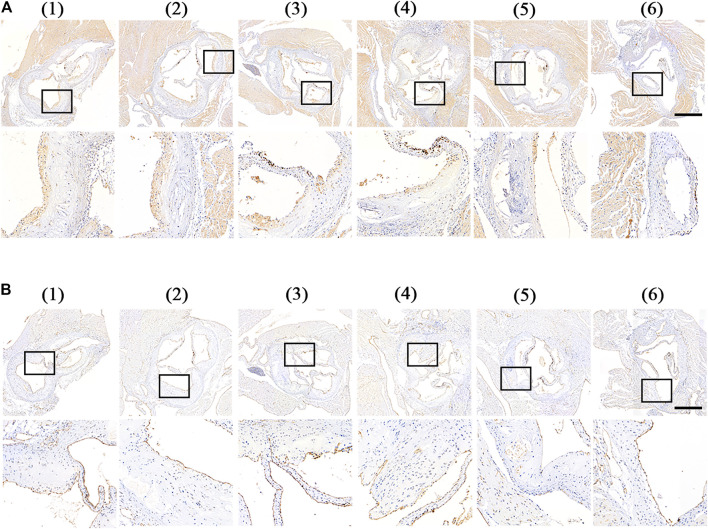
Immunohistochemistry staining of aortic root sections from ApoE^−/-^ mice after different treatment at day 30 (Wang et al., 2021).- (Costagliola di Polidoro et al., 2021) referred to (Wang et al., 2021) PBS (Libby, 2012), free RAP (Shapiro and Fazio, 2016), RAP@NPs (Moriya, 2019), RAP@PLT NPs (Winkel et al., 2014), RAP@NPs + SonoVue™ + US (Costagliola di Polidoro et al., 2021), RAP@PLT NPs + SonoVue™ + US. Representative images of immunohistochemistry staining with antibodies to **(A)** MMP-9 and **(B)** CD31 (scale bar = 500 µm).

Furthermore, we analyzed the components of atherosclerotic plaques in aortic root sections by immunohistochemistry staining. The rupture of the plaque is mainly caused by the rupture of the fiber cap on the surface of the plaque. The rupture of the fibrous cap is mainly caused by matrix metalloproteinases (MMPs) produced by foam cells in the plaque. MMPs can degrade collagen and destroy the fibrous cap. Therefore, the expression of MMPs in the plaque can reflect the stability of the plaque to a certain extent (Galis and Khatri, 2002). As shown in Figure 8A, a large amount of MMP-9 was distributed at the plaque in the control group. After 1-month of treatment, MMP-9 was obviously decreased in the free RAP, RAP@NPs, and RAP@PLT NPs groups. The inhibition was most significant in the RAP@PLT NPs + SonoVue™ + US group.

Vascular endothelial cells play an important role in the homeostasis of arteries, and are a natural mechanical barrier with a modulating effect. To detect the condition of vascular endothelial cells at the root of aortic arch in mice after treatment, mouse aortic arch root sections were subjected to CD31 (endothelial cell marker) immunohistochemical analysis. As shown in Figure 8B, in the plaques of the control group, free RAP and RAP@NPs groups, vascular endothelial CD31 expression was interrupted, while in the RAP@NPs + SonoVue™ + US and RAP@PLT NPs + SonoVue™ + US groups, the vascular endothelium continuously expressed CD31, indicating that the RAP@PLT NPs + SonoVue™ + US treatment group can better maintain the integrity of the vascular endothelium.

**FIGURE 9:**
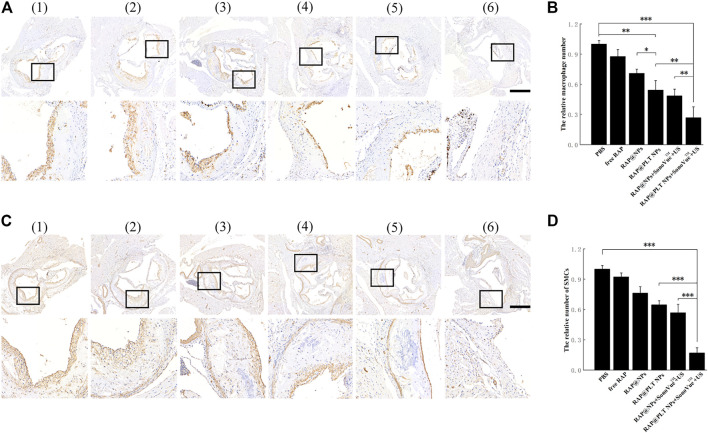
Immunohistochemistry staining of aortic root sections from ApoE^−/-^ mice after different treatment at day 30 (Wang et al., 2021).- (Costagliola di Polidoro et al., 2021) referred to (Wang et al., 2021) PBS (Libby, 2012),free RAP (Shapiro and Fazio, 2016),RAP@NPs (Moriya, 2019),RAP@PLT NPs (Winkel et al., 2014), RAP@NPs + SonoVue™ + US (Costagliola di Polidoro et al., 2021), RAP@PLT NPs + SonoVue™ + US. Representative images of immunohistochemistry staining with antibodies to **(A)** CD68 and **(C)** α-SMA (scale bar = 500 µm). Quantitative data of **(B)** the relative number of macrophages and **(D)** SMCs in plaque areas of the aortic root sections. *n* = 5, mean ± SD, *p < 0.05, **p < 0.01, and ***p < 0.001.

Macrophages play an irreplacable role in the occurrence and development of atherosclerotic plaques. We conducted CD68 (macrophage marker) immunohistochemical analysis on mouse aortic root slices after different treatments. As shown in Figure 9A, a large number of macrophages was present in the plaques of the control group, while the number gradually decreased in the free RAP, RAP@NPs and RAP@PLT NPs groups. After addition of the SonoVue™ + US treatment, the number of macrophages in the plaques decreased sharply in both the RAP@NPs + SonoVue™ + US and RAP@PLT NPs + SonoVue™ + US groups. The results were further confirmed by quantitative analysis (Figure 9B).

During the development of AS, SMCs become abnormal under the stimulation of various factors and promote the formation of atherosclerotic plaques. *α*-SMA (smooth muscle cell marker) immunohistochemical analysis of aortic arch root slices of mice was carried out, as shown in Figure 9C. Many SMCs were distributed in the plaques of the control group. As shown in Figure 9D, the number of SMCs in the plaques in the free RAP, RAP@NPs and RAP@PLT NPs gradually decreased, while the number of SMCs in the plaques decreased sharply in the groups treated with SonoVue™ + US. The number of SMCs in the plaque of the RAP@PLT NPs + SonoVue™ + US group was only 16.95% of that of the control group. In summary, with the treatment of RAP@PLT NPs + SonoVue™ + US, the number of SMCs and macrophages decreased, proving that this scheme could efficiently inhibit the proliferation of SMCs and macrophages in plaques, which is consistent with previous experimental results.

### Biosafety Assessment

**FIGURE 10:**
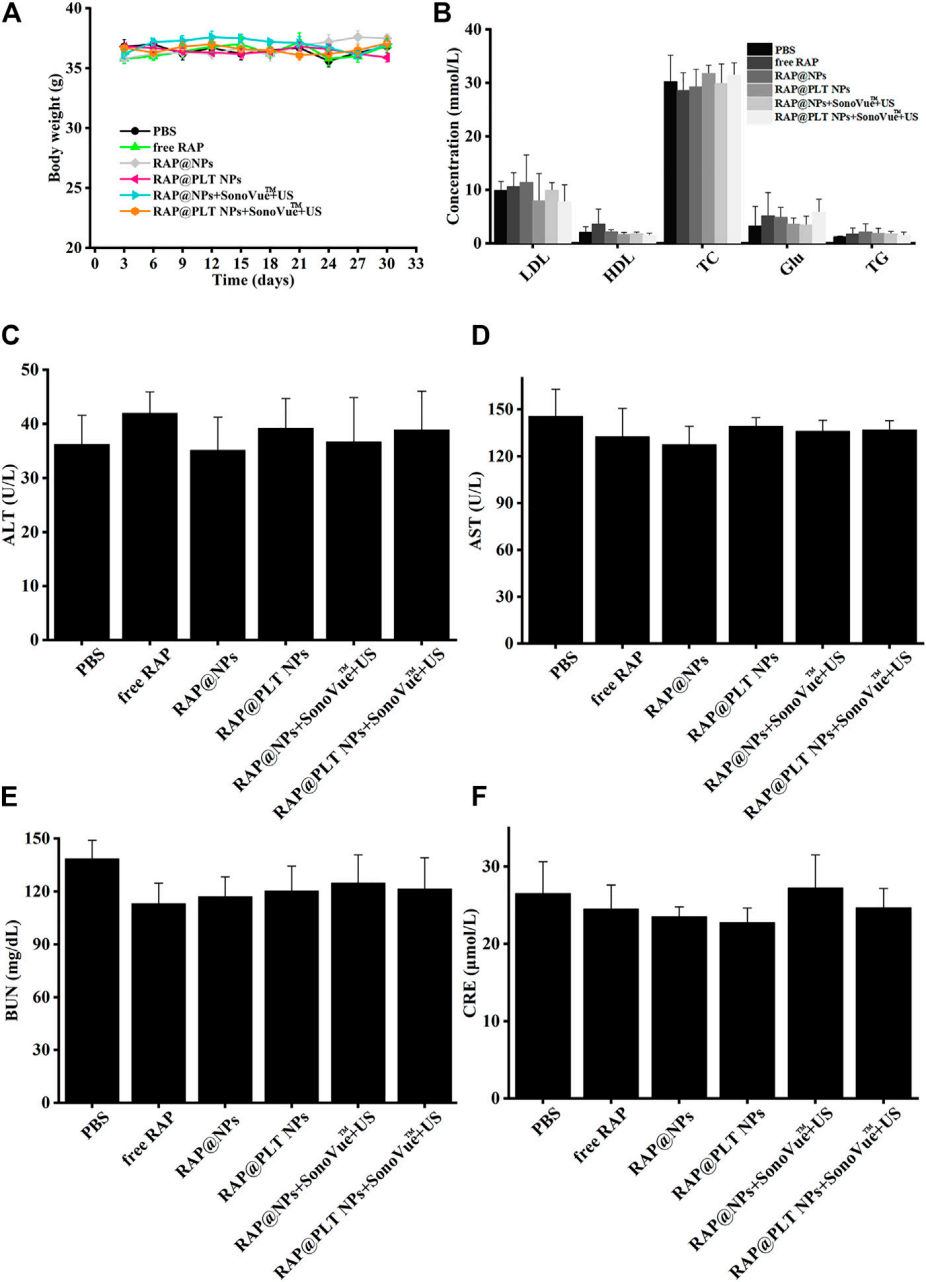
Biosafety analysis of ApoE^−/-^ mice after different treatment at day 30. **(A)** The body weight of ApoE^−/−^mice during various treatments. **(B)** The lipid profile of ApoE^−/-^ mice after different treatment. **(C–F)** The biochemical assays of hepatic and kidney functions. ALT, alanine aminotransferase; AST, aspartate aminotransferase; URN, blood urea nitrogen; and CRE, creatinine. *n* = 5, mean ± SD.

**FIGURE 11:**
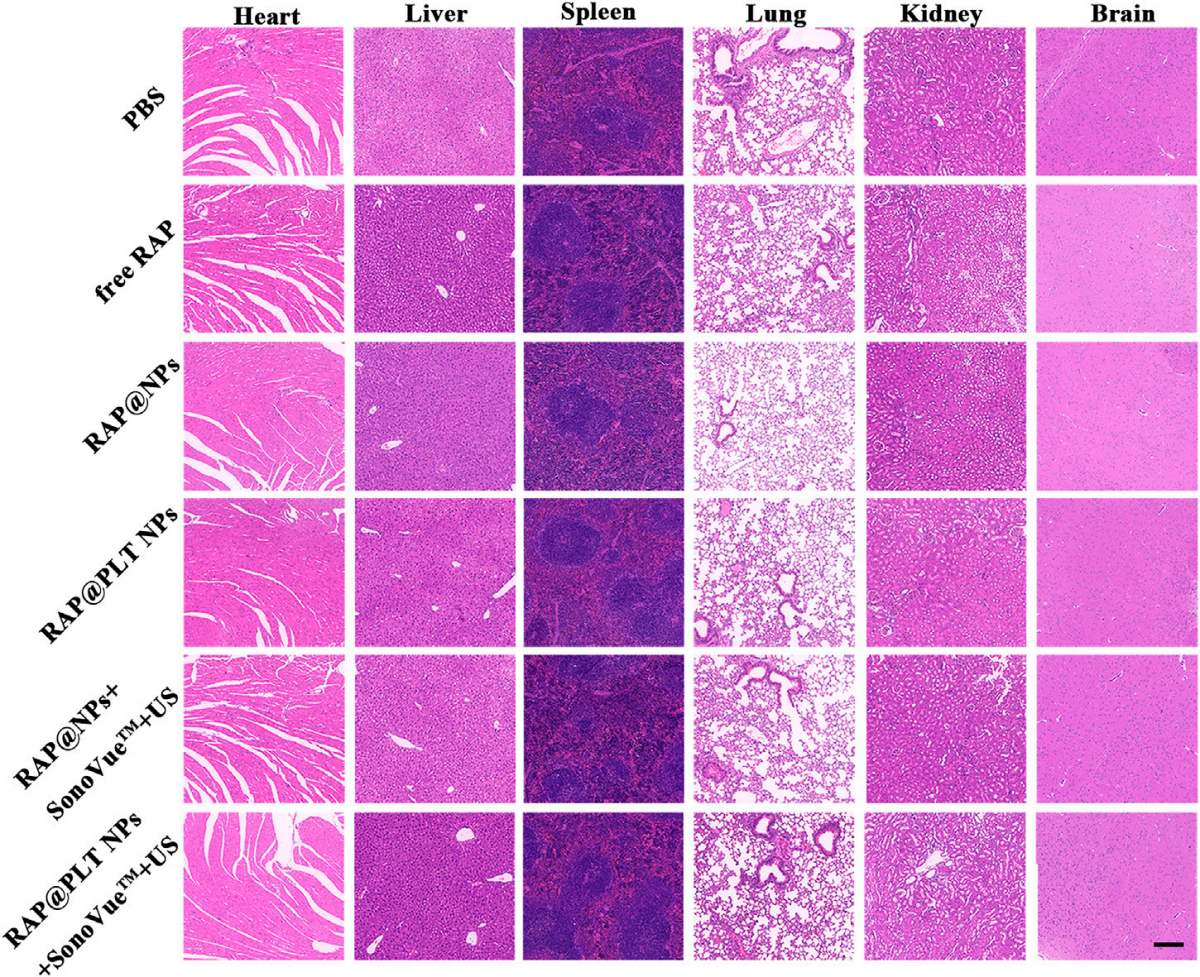
Histological observation of the organs collected from the ApoE^−/-^ mice after the treatment at day 30. The organ sections were stained with H&E. Scale bar: 200 µm.

To evaluate the safety of treatment, we recorded the body weight of ApoE^−/−^ mice and detected serological indicators, as well as HE staining of major organs, after 1 month of treatment. All mice survived at the end of treatment, and the body weight of the mice in each group did not decrease significantly, as shown in Figure 10A, suggesting that RAP@PLT NPs + SonoVue™ + US had no obvious side effects on ApoE^−/−^ mice. At the end of the treatment, we also collected whole blood from mice from each group to analyze the lipid profile and blood biochemistry. RAP could theoretically cause dyslipidemia, while ApoE^−/-^ mice may also have lipid abnormalities during a high-fat diet. In our final results, as shown in Figure 10B, there was no significant difference in the levels of serum lipids, including LDL, TG, TC and HDL between the different groups. As shown in Figures 10C–F, there was also no obvious difference in the levels of serum ALT, ASL, BUN and CRE, indicating that the nanoparticle formulations with SonoVue™ + US treatment we used were relatively safe. In the groups treated with RAP@PLT NPs + SonoVue™ + US, there were no obvious pathological changes in the H&E-stained sections of major organs compared with the PBS group (Figure 11). In conclusion, the biomimetic RAP@PLT NPs + SonoVue™ + US treatment program shows good biocompatibility.

## Conclusion

Cell membrane coating technology is a promising strategy for targeted therapy, and UTMD is a very promising supporting method for targeted drug delivery therapy. In this study, we studied RAP@PLT NPs with the assistance of SonoVue™ + US, which could increase the targeting ability of nanoparticles to atherosclerotic plaques by improving the efficiency of RAP release and the destruction of neovascularization in the plaques. Therefore, the stability of vulnerable plaques improves the therapeutic effect of RAP on plaques. In addition, this treatment is relatively safe. Overall, this study emphasizes the great potential of UTMD in promoting targeted nanoparticles to enter blood vessels and plaques, which is very promising for the treatment of cardiovascular diseases.

## Data Availability

The raw data supporting the conclusion of this article will be made available by the authors, without undue reservation.
