# Supplementary material for: Platelet Membrane Biomimetic Nanoparticles Combined With UTMD to Improve the Stability of Atherosclerotic Plaques
Source: Front Chem. 2022 Mar 8;10:868063. doi: 10.3389/fchem.2022.868063 (PMC8958035; doi:10.3389/fchem.2022.868063)
Supplement: Supplementary file 1 [file DataSheet1.docx]

Platelet membrane biomimetic nanoparticles combined with ultrasonic cavitation effect to improve the stability of atherosclerotic plaque

Jia Zhou^1,2#^, Chengcheng Niu^3#^, Biying Huang^3^, Sijie Chen^3^, Caigui Yu^1^, Sheng Cao^1*^, Wenjing Pei^3*^, Ruiqiang Guo^1*^

**^1^** Department of Ultrasound Imaging, Renmin Hospital of Wuhan University, Wuhan, Hubei 430060, China

**^2^** Department of Ultrasound Medicine, The First Affiliated Hospital, Hengyang Medical School, University of South China, Hengyang, Hunan 421001, China.

**^3^** Department of Ultrasound Diagnosis, The Second Xiangya Hospital, Central South University, Changsha, Hunan 410011, China

Address all correspondence to:

Sheng Cao, E-mail: [caosheng209@126.com](mailto:caosheng209@126.com); Department of Ultrasound Imaging, Renmin Hospital of Wuhan University, Wuhan, Hunan, China, 430060;

Wenjing Pei, E-mail: [459315053@qq.com](mailto:459315053@qq.com); Department of Ultrasound Diagnosis, The Second Xiangya Hospital, Central South University, Changsha, Hunan, China, 410011;

Ruiqiang Guo, E-mail: guorq.wh.edu@hotmail.com; Department of Ultrasound Imaging, Renmin Hospital of Wuhan University, Wuhan, Hunan, China, 430060.

^#^ These authors have contributed equally to this work.

**Methods**

***The preparation of platelet membranes***

To isolate platelets, the mice whole blood was first centrifuged at 300 × g for 5 minutes at room temperature. The supernatant is then collected and centrifuged at 300 × g for another 5 minutes. The supernatant obtained is platelet rich plasma (PRP). Afterwards, the PRP was centrifuged at 2000 × g for 4 minutes in order to precipitate the platelets. The platelets membrane was obtained by repeated freeze–thaw process. Aliquots of PLT suspensions were frozen at −80 °C, and thawed at room temperature. After repeated three times, the pellet was washed with PBS at 2,1000 × g for three times and finally resuspended in water, sonicated for 5 minutes and stored at -80 °C.

### *Preparation of RAP@NPs*

The RAP@NPs were fabricated using a published method with minor modifications. First, 100 mg of PLGA and a suitable amount of RAP were added to 3 mL of chloroform and stirred to completely dissolve; then, 15 mL of cold PVA solution (5% w/v) was added to the above system. The mixture was emulsified with an ultrasonic processor for 90s, stirred and evaporated at room temperature for2~5 h, and then washed with phosphate-buffered saline (PBS) several times.

***Preparation of DiI labeled nanoparticles***

The DiI labeled nanoparticles were prepared as the following procedure. First, 100 mg of PLGA, a suitable amount of DiI were added to 3 mL of chloroform and stirred to completely dissolve; then, 15 mL of cold PVA solution (5% w/v) was added to the above system. The mixture was emulsified with an ultrasonic processor for 90s, stirred and evaporated at room temperature for2~5 h, and then washed with phosphate-buffered saline (PBS) several times. The DiI labeled-nanoparticles (DiI@PLGA NPs) were collected.

Then, 1 mL of 1 × PBS containing 1 mg of DiI labeled PLGA NPs was mixed with the PLT vesicles and sonicated for 30 s to complete the membrane coating. The collected DiI labeled PLT-DiI@PLGA NPs were redispersed in 1 × PBS and kept at 4 °C.

***Preparation of IR780 labeled nanoparticles***

The IR780 labeled nanoparticles were prepared as the following procedure. First, 100 mg of PLGA, a suitable amount of RAP and 3 mg of IR780 were added to 3 mL of chloroform and stirred to completely dissolve; then, 15 mL of cold PVA solution (5% w/v) was added to the above system. The mixture was emulsified with an ultrasonic processor for 90s, stirred and evaporated at room temperature for2~5 h, and then washed with phosphate-buffered saline (PBS) several times. The IR780 labeled-RAP@NPs were collected.

Then, 1 mL of 1 × PBS containing 1 mg of IR780 labeled RAP@NPs was mixed with the PLT vesicles and sonicated for 30 s to complete the membrane coating. The collected IR780 labeled RAP@PLT NPs were redispersed in 1 × PBS and kept at 4 °C.

### *Nanoparticles Uptake by Macrophages*

RAW264.7 cells were seeded in 12-well plates at a density of 1 × 10^5^ cells per well in 1 mL of DMEM containing 10% FBS and cultured at 37 °C with 5% CO_2_ for 24 h. DiI@PLGA NPs and PLT-DiI@PLGA NPs were separately added and incubated for 2 h. Then, the cells were gently washed with PBS and fixed with 4% paraformaldehyde. The nuclei of the cells were stained with DAPI. The cells were observed using an inverted fluorescence microscope. For quantitative analysis, the gray value of images with red fluorescence was analyzed by ImageJ.

***Accumulation of RAP@PLT NPs in Atherosclerotic Plaques***

To further study the atherosclerotic plaque targeting ability of RAP@PLT NPs, 200 µL of IR780@PLT NPs, IR780@PLT NPs and IR780@PLT NPs + SonoVue^TM^ was administered to atherosclerosis model mice through the tail vein, and then those mice given IR780@PLT NPs + SonoVue^TM^ were irradiated with ultrasound (2 W/cm^2^) at the chest area for 30 s. After 2 h, mice were euthanized, and perfused with PBS containing 4% paraformaldehyde and heparin sodium, and the aorta was isolated for imaging and fluorescence quantification using the former Lumina IVIS Spectrum imaging system.

### *Complete Blood Biochemistry and Routine Analysis*

An EDTA spray-coated tube was used to collect the blood of each group at the end of treatment and immediately analyzed with an automatic hematology analyzer (Mindray BS-430, Mindray Co., China) for serum lipids and blood glucose to obtain the following parameters: total cholesterol (TC), triglyceride (TG), high-density lipoprotein (HDL), and low-density lipoprotein (LDL). Hematological parameters such as alanine aminotransferase (ALT), aspartate aminotransferase (AST), creatinine (CREA) and blood urea nitrogen (UREA) were also quantified.





**Figure S1** Size change tendency of RAP@PLT NPs (n = 3).


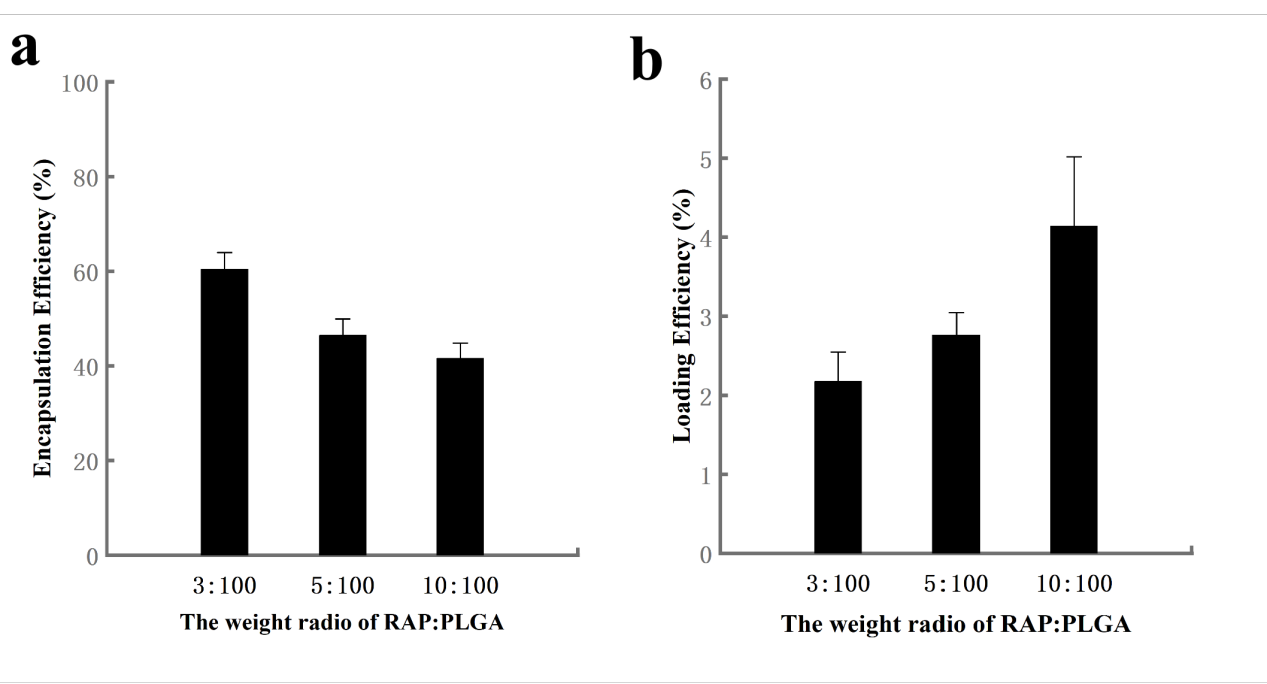


**Figure S2** (a) RAP encapsulation efficiency and (b) loading efficiency of RAP@NPs with different input weight radio of RAP:PLGA(3:100,5:100,10:100).





**Figure S3** Drug release profile of RAP@NPs and RAP@PLT NPs with or without US in 72h (n = 3).





**Figure S4** *In vitro* cytotoxicity of free RAP, RAP@NPs, and RAP@PLT NPs after incubation for 24 h.


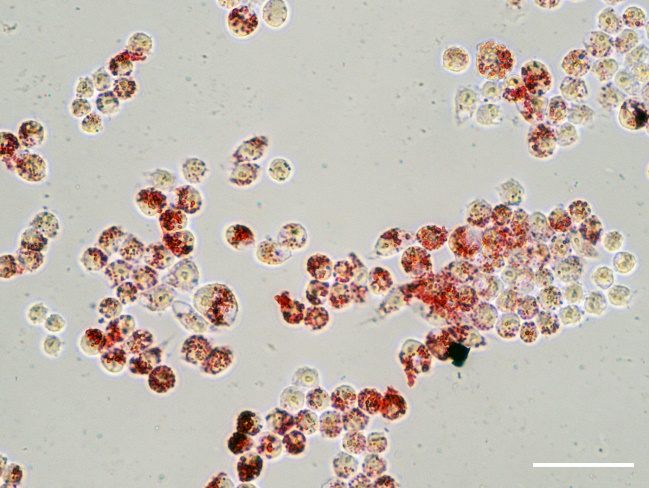


**Figure S5** Foam cells that were induced from Raw 264.7 murine macrophages stained with Oil Red O, and observed with an inverted microscope (scale bar = 50 µm).





**Figure S6** Pharmacokinetics of IR780-labelled RAP@NPs and RAP@PLT NPs after intravenous injection via tail vein at IR780 dose of 0.5 mg/kg. The plasma IR780 concentration curves of RAP@ NPs and RAP@PLT NPs.

**
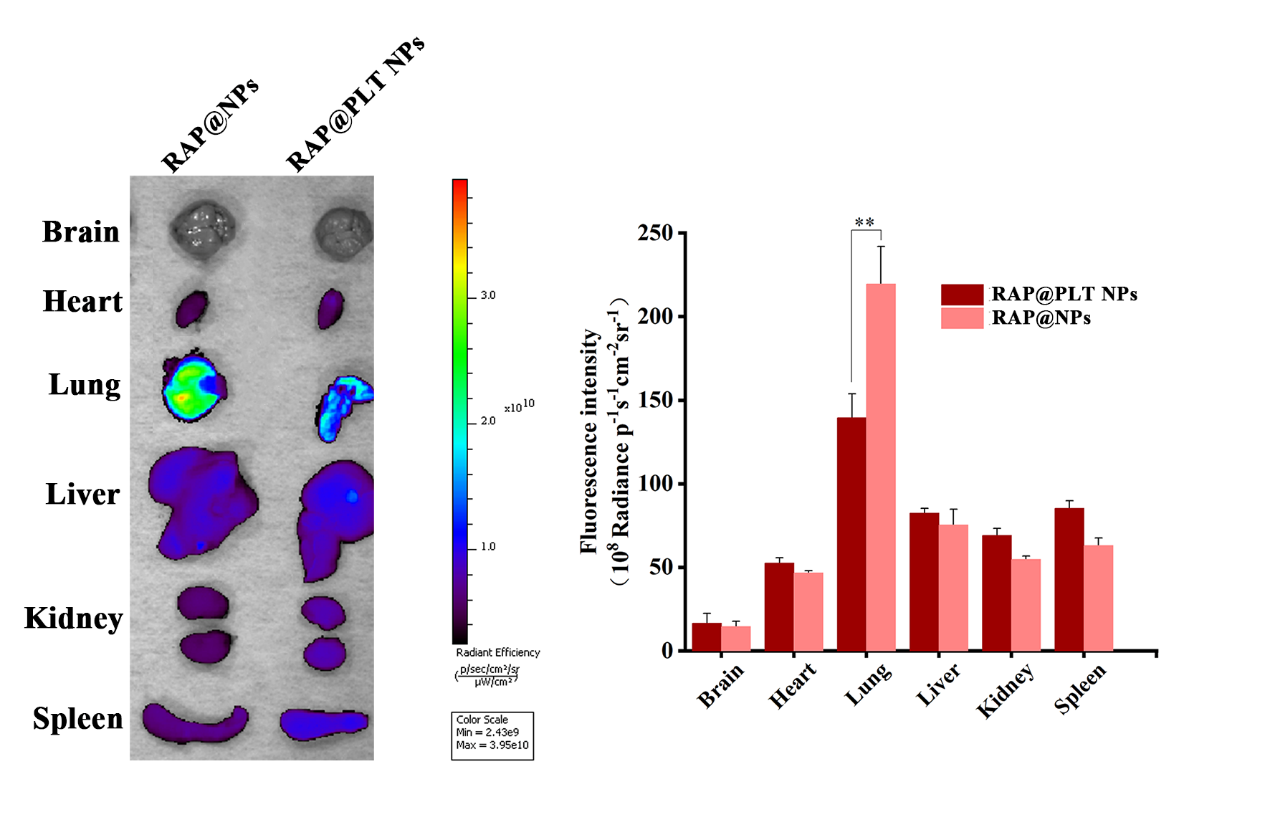
**

**Figure S7.** *In vivo* distribution of RAP@PLT NPs in atherosclerotic ApoE ^-/-^ mice. Twenty-four hours after injection, major organs were harvested and imagined (Left panel) and the mean fluorescence intensity in major organs were quantified (Right panel)

**Table. S1** Pharmacokinetic parameters of IR780-labelled RAP@NPs and RAP@PLT NPs.

| Parameter | RAP@ NPs | RAP@PLT NPs |
| --- | --- | --- |
| AUC (μg/mL·h)_0-t_ | 88.389±7.41 | 116.996±7.15** |
| T_1/2_ (h)  CL (mL/h) | 10.330±1.25  0.170±0.02 | 13.428±2.61**  0.128±0.01* |

AUC_0-t_: Area under plasma IR780 concentration versus time curves

T_1/2_: Elimination half-life

CL: in vivo clearance rate

*P˂0.05, **P˂0.01 (two-tailed Student’s t-test).
